# The Sonographic Evaluation of Abductor Injury After Intramedullary Nailing for the Hip Fractures

**DOI:** 10.3390/jcm14155498

**Published:** 2025-08-05

**Authors:** Yonghyun Yoon, Howon Lee, King Hei Stanley Lam, Minjae Lee, Jonghyeok Lee, Jihyo Hwang

**Affiliations:** 1 Department of Orthopedic Surgery, Gangnam Sacred Heart Hospital, Hallym University College of Medicine, 1 Singil-ro, Yeongdeungpo-gu, Seoul 07441, Republic of Korea; mgyyh00@gmail.com (Y.Y.); ehw80@hallym.or.kr (H.L.); 2 Department of Orthopaedic Surgery, Incheon Terminal Orthopaedic Surgery Clinic, 5F, 4, 489 Inha-ro, Namdong-gu, Incheon 21574, Gyeonggi, Republic of Korea; mjlee951224@gmail.com (M.L.); perfectceive@gmail.com (J.L.); 3 International Academy of Regenerative Medicine, 4F, 4, 489 Inha-ro, Namdong-gu, Incheon 21574, Gyeonggi, Republic of Korea; drlamkh@gmail.com; 4 The Board of Clinical Research, The International Association of Musculoskeletal Medicine, Kowloon, Hong Kong; 5 Faculty of Medicine, The University of Hong Kong, Kowloon, Hong Kong; 6 Faculty of Medicine, The Chinese University of Hong Kong, New Territory, Hong Kong; 7 The Board of Clinical Research, The Hong Kong Institute of Musculoskeletal Medicine, Kowloon, Hong Kong; 8 Bareun Neurosurgery Clinic, 39, Daenong-ro, Heungdeok-gu, Cheongju-si 28402, Chungcheongbuk-do, Republic of Korea

**Keywords:** sonography, abductor tendons, intramedullary nailing, hip fractures

## Abstract

**Background/Objectives:** Iatrogenic abductor muscle injury following intramedullary nailing for proximal hip fractures can negatively impact postoperative rehabilitation and clinical outcomes. To quantify iatrogenic abductor muscle injury after intramedullary nailing and detect the degree of degenerative change in muscle around the entry point of trochanteric fractures. **Methods:** This cross-sectional study used data from a single center database from May to December 2023. This study utilized ultrasound examinations performed by a single expert orthopedic surgeon. This study included 61 patients who underwent intramedullary nailing surgery for adult hip fractures. All surgeries were performed by a single experienced hip surgeon. Patients who declined sonographic evaluation or did not undergo ultrasound during their admission were excluded. For more accurate comparison, sonography was also conducted on the healthy, non-operative limb. Descriptive statistics were used to summarize patient and ultrasound findings. A subgroup analysis using Fisher’s exact test was performed to assess the association between implant type and the incidence of iatrogenic gluteus medius tendon injury. **Results:** Of the 61 patients, tendon tears were identified in 35 cases (57%) on the affected side, with 20 cases (33%) involving gluteus medius tendon tears without fractures on the ipsilateral facet. Gluteus minimus tendon tears were observed in 13 cases (21%), while gluteus medius tendon tears were noted in 31 cases (51%). In the unaffected limbs, tendon degeneration was detected in the form of tendinosis and calcification. Overall, 39 patients (64%) exhibited abductor tendon tendinosis, and 30 patients (49%) were diagnosed with calcification. **Conclusions:** Gluteus medius and Gluteus minimus are important abductors for hip disease rehabilitation. Iatrogenic gluteus medius tendon injury during the intramedullary nailing showed 33%. Abductor degeneration also showed 92% of the unaffected limbs. This study suggests that abductor degeneration can be a risk factor of falling among the elderly population and an iatrogenic abductor injury can be an obstacle for the early recovery of ambulation in the hip fracture patients. Prevention of abductor degeneration and iatrogenic abductor injury might be important for the hip fracture prevention and rehabilitation.

## 1. Introduction

Hip fractures are pathological fractures with a high incidence among the elderly worldwide. If left untreated, more than 50% of patients die within a year [1], and even with surgical treatment, approximately 20% of patients die within a year, making it a life-threatening type of fracture [2,3]. Due to their anatomical characteristics, proximal hip fractures are the most common type of fracture, and the gold standard treatment for extracapsular fractures is intramedullary nailing (IM nailing) [4]. IM nailing is widely used due to its short surgical duration and its ability to facilitate early ambulation following treatment [5,6].

However, in clinical practice, some patients undergoing IM nailing fail to initiate early ambulation, suggesting potential damage to the muscles involved in gait. Indeed, concerns have been raised that the gluteus medius (Gme) may be injured during the process of positioning the patient on the fracture table and accessing the greater trochanteric (GT) fossa from the lateral side to insert the IM nail [7,8,9,10,11].

The Gme which covers the GT fossa like an umbrella, attaches to the lateral (L) and posterior (P) facets of greater trochanter [12,13], while the gluteus minimus (Gmi) originates from the iliac crest and attaches to the anterior (A) facet of the GT [14]. These gluteal muscles, in particular, are one of the muscles with large cross-sectional area (CSA) among those attached to the hip joint and plays a crucial role in ambulation [15].

When these muscles attached to the GT are chronically affected, patients may experience walking difficulties and lower back pain, a condition collectively referred to as greater trochanteric pain syndrome (GTPS) [13,16,17]. Conservative treatment options for GTPS include NSAIDs, physical therapy, steroid injections, extracorporeal shockwave therapy (ESWT), prolotherapy, and platelet-rich plasma (PRP) injections [18,19,20,21,22]. Surgical treatments, such as arthroscopic debridement, repair, or open surgery, are considered when conservative treatments fail to alleviate symptoms [23,24,25].

**Figure 1:**
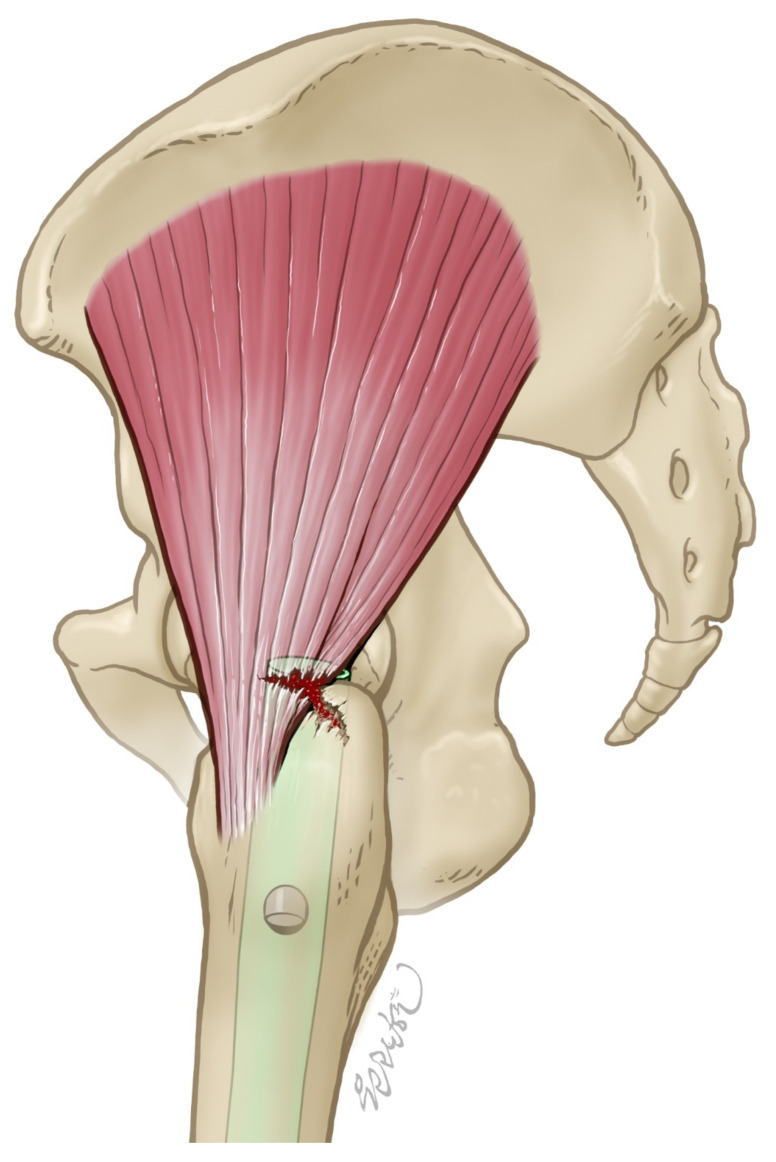
This is a picture depicting an abductor injury that occurs during intramedullary nailing or fracture.

While chronic conditions like GTPS are well-studied, with gluteal muscle injuries frequently evaluated using ultrasound [26,27], there has been no research utilizing ultrasound for acute or iatrogenic injuries to the gluteal muscles following hip fractures and surgical interventions, such as IM nailing (Figure 1). Fractures are often accompanied by acute injuries to surrounding ligaments and muscles [28,29], yet ultrasound assessments of these injuries, particularly in the setting of proximal hip fractures, remain unexplored. This study aims to address this gap by assessing the incidence of acute hip abductor injury that occurs during IM nailing and verifying the extent of gluteal muscle injuries using ultrasound, comparing the injured side to the unaffected hip joint. This investigation highlights the need for targeted postoperative rehabilitation that considers potential acute muscle injuries.

## 2. Materials and Methods

### 2.1. Study Design

This cross-sectional study was conducted at the Department of Orthopedic Surgery, Hallym University Gangnam Sacred Heart Hospital, between May 2023 and December 2024. Patients who were hospitalized for proximal hip or femoral fractures and underwent intramedullary (IM) nailing, as well as patients who underwent IM nail removal related to prior hip fracture treatment, were screened for eligibility.

Inclusion criteria were as follows: (1) Patients aged ≥ 50 years; (2) Patients who sustained a unilateral proximal femoral or hip fracture and were treated with intramedullary nailing. All surgeries were performed by a single orthopedic surgeon (JHH).

Exclusion criteria were as follows: (1) patients with bilateral fractures or fractures at other anatomical sites; (2) patients who were unable to undergo ultrasound examination due to medical instability, poor cooperation, or refusal to participate in the study.

### 2.2. Ethics Approval

This study was conducted on 61 patients who underwent IM nailing for adult hip fractures between May and December 2023. Ethical approval for this study was obtained from the Institutional Review Board of Hallym University Gangnam Sacred Heart Hospital (No. 2022-12-004-007), and the study was carried out in accordance with the Declaration of Helsinki.

### 2.3. Patient Characteristics

The average age of the patients was 79 years (SD, 12), consisting of 15 males and 46 females. The diagnoses included trochanteric fractures in 48 patients (79%), mid-shaft fractures in 5 patients (8%), subtrochanteric fractures in 4 patients (7%), femur neck stress fracture in 1 patient (2%), proximal shaft fracture in 1 patient (2%), mid-shaft peri-implant fracture in 1 patient (2%), and IM nail removal in 1 patient (2%). The mid-shaft fractures were included in the study as they also require IM nailing. Fractures were diagnosed and categorized using standard orthopedic classification systems appropriate to each fracture type. Femoral neck fractures were classified using the Garden classification system [30]. Intertrochanteric and subtrochanteric fractures were classified according to the AO/OTA classification (31A1–31A3), which provides practical reproducibility and has demonstrated acceptable reliability in previous studies [31,32]. Femoral shaft fractures were also classified using the revised AO/OTA classification system, as validated for mid-diaphyseal injuries [33]. The case of IM nail removal cases was included in evaluating potential iatrogenic injury of gluteus muscle and its subsequent rehabilitation [34] (Table 1).

### 2.4. Surgical Intervention

IM nailing procedure was performed under general anesthesia on a fracture table. The patient was positioned in the supine position with the unaffected leg maximally abducted, then secured to the table to facilitate the acquisition of lateral imaging [35]. In the single case of IM nail removal, the procedure was performed with the patient in the lateral position. Traction and rotation were applied to perform a closed reduction [36,37]. In cases where closed reduction was difficult or lost during surgery, a minimal incision was made at the fracture site to expose subcutaneous tissue and muscles. Traction devices and bone hooks were then used to achieve reduction. The skin overlying the apex of the greater trochanter was incised, and the guidewire was accurately positioned at the GT fossa, which served as the nail’s entry point. A reamer was then used to enlarge the medullary canal, followed by the insertion of the intramedullary nail. Various types of intramedullary nails were used depending on the fracture pattern and surgical plan. These included the GS Hip Nail (n = 43; GS Medical, Cheongwon-gun, Republic of Korea), Gamma3 U-blade Nail (n = 9; Stryker, Duisburg, Germany), Gamma3 Long Nail (n = 7; Stryker, Duisburg, Germany), and Zimmer Natural Nail (n = 1; Zimmer-Biomet, Warsaw, IN, USA). In one case corresponding to the IM nail removal described above, the Proximal Femoral Nail Antirotation (PFNA; Synthes, Solothurn, Switzerland) had been inserted previously and was removed during the procedure, rather than being implanted as part of this study. All surgeries were performed by one experienced surgeon.

### 2.5. Ultrasound

Ultrasound examination and interpretation were performed by an orthopedic specialist with more than 10 years of experience in musculoskeletal ultrasound and procedures. To account for potential errors due to degenerative changes when evaluating only the affected side, the unaffected side was also assessed on the same day of the examination.

Ultrasound evaluations were performed on average post-operative day (POD) of 6.2 (range, POD1-POD20) in the lateral decubitus position. If the lateral decubitus position was not feasible due to post-operative pain or other factors, the examination was performed in the supine position. The unaffected side was assessed in the same manner as the affected side.

Ultrasound was performed using the HS60 machine with a linear probe of 2–8 MHz, a setting of 5–6 cm and a focus position on the greater trochanter. For the unaffected side, since there was no swelling unlike the surgical site, the depth and focus settings were adjusted accordingly.

**Figure 2:**
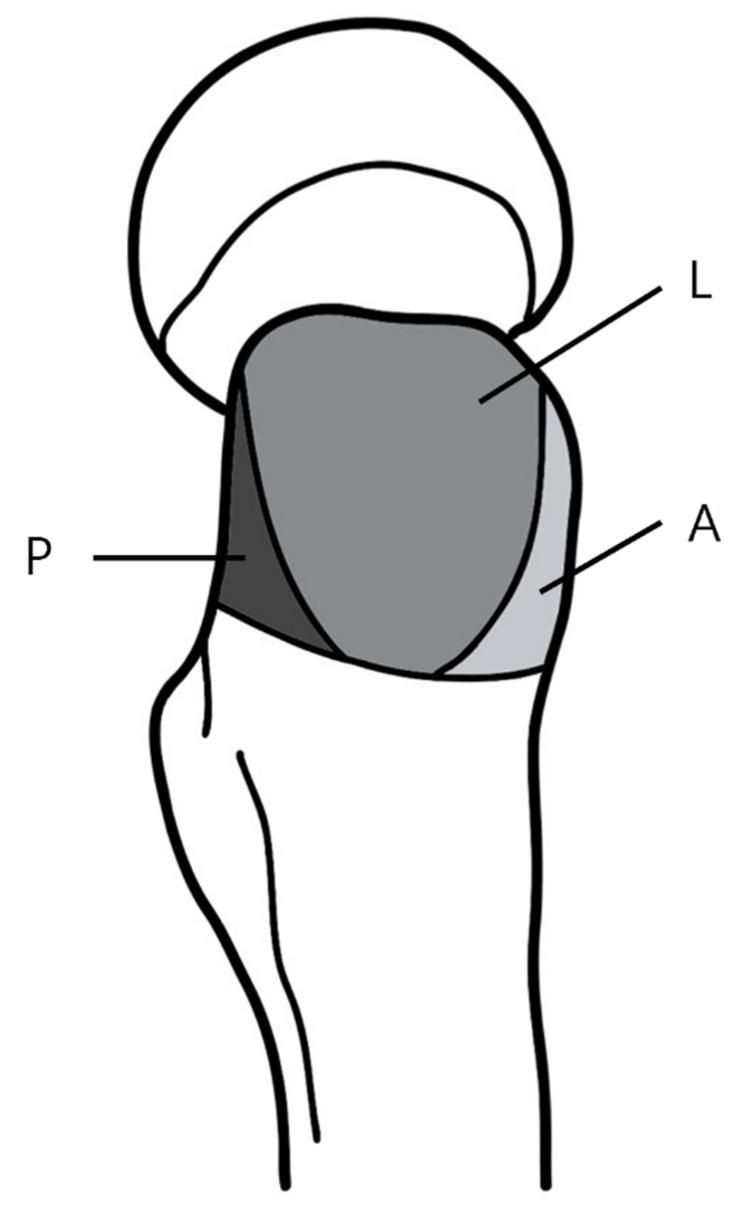
Three facets of greater trochanter. A is the anterior facet, L is the lateral facet, P is the posterior facet.

**Figure 3:**
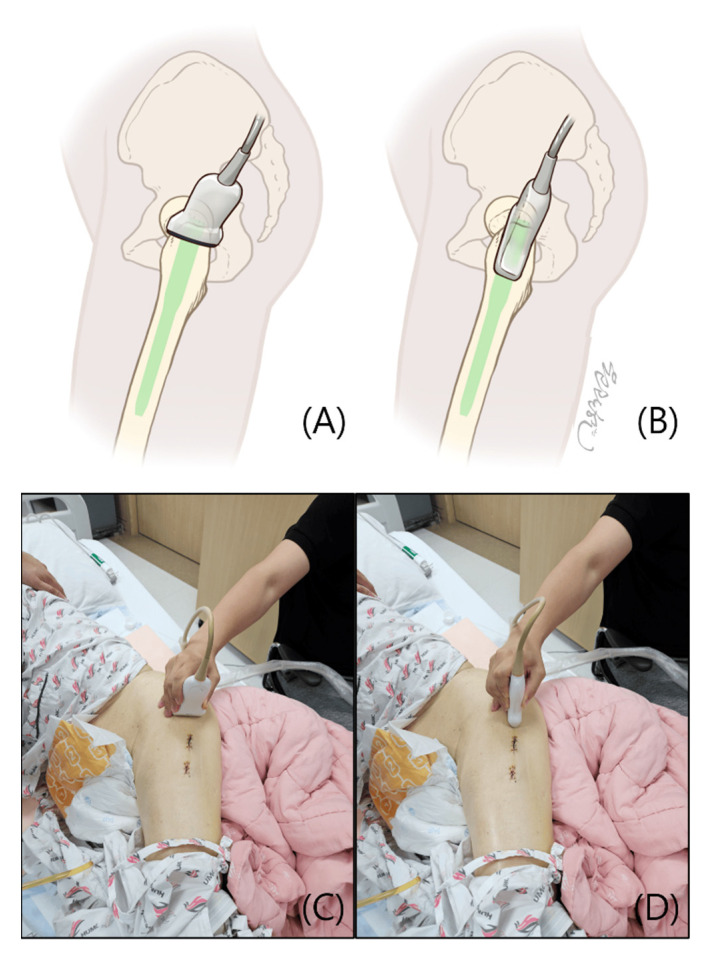
To perform ultrasound, the patient was first positioned in the lateral decubitus position. The hip on the side to be examined was flexed to 15–30° and the knee was flexed to 30°. (**A**,**C**) In the short-axis (SAX) view, the transducer was placed on the apex of the GT in a transverse plane perpendicular to the femoral shaft. (**B**,**D**) In the long-axis (LAX) view, the transducer was placed on the apex of the GT in a plane parallel to the femoral shaft.

Ultrasound evaluation was performed by dividing the GT in three areas: the anterior facet where Gmi attaches, the lateral and posterior facets where the Gme attaches (Figure 2). To improve diagnostic accuracy and minimize the volume averaging effect, both short-axis (SAX) and long-axis (LAX) views were assessed [38] (Figure 3).

**Figure 4:**
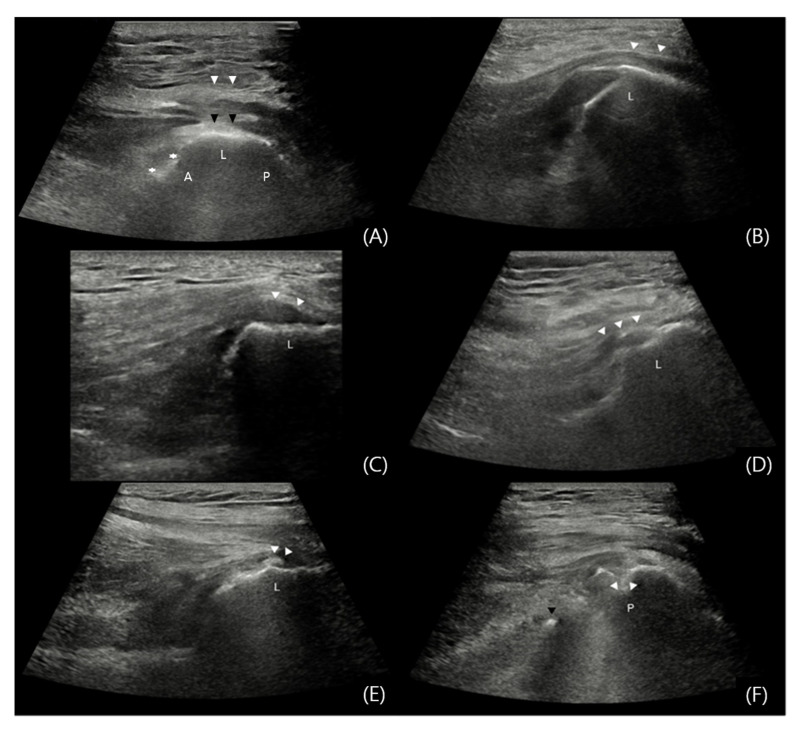
Ultrasound Assessment of the Gluteal Muscles. When evaluating in the SAX view, the transducer was positioned transversely to the long axis of the femur and moved from proximal to distal until the peak of GT, composed of three facets (A, L, and P) was visualized. The A facet is attached to the Gmi, while the L and P facets are attached to the Gme. Once the GT appears in the SAX view, the transducer is rotated 90° to switch to the LAX view, then moved anteriorly, parallel to the femur. (**A**) Normal: In the SAX view, the Gmi tendon (asterisks) is seen attached to the A facet, while the Gme (black arrows) covering the L and P facets, with the iliotibial (IT) band (white arrows) visible above. (**B**) Normal: In the LAX view of the L facet, the Gme tendon (white arrows) can be distinguished from the Gmi as it extends distally beyond the facet, attaching to the distal portion. (**C**) Tendinosis: Thickening of the hypoechoic fibrillar portion of the Gme tendon (white arrows) is observed. (**D**) Tendon Tear: In the LAX view of the L facet, a hypoechoic and fibrillar disruption lesion (white arrows) greater than 25% of the Gme tendon height, is visible. (**E**) Calcification: In the LAX view of the L facet, hyperechoic foci (white arrows) in the Gme tendon area are seen. (**F**) Fracture: In the LAX view of the P facet, areas where the continuity of the bony contour is disrupted (white arrows) are observed. The tip of the inserted nail, protruding beyond the GT fossa, is visualized as a hyperechoic lesion (black arrow) on ultrasound.

**Figure 5:**
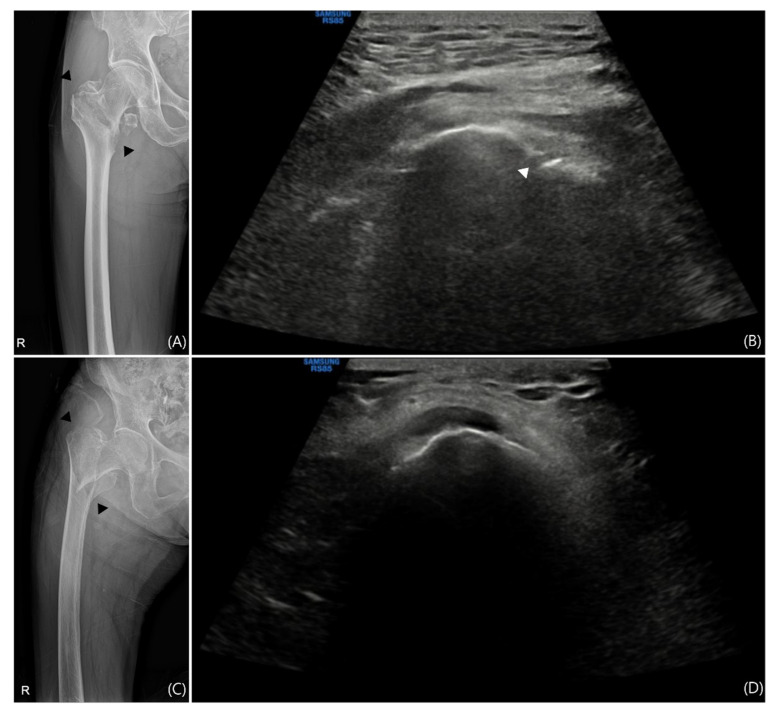
Comparison of X-ray and Ultrasound in Patients with Trochanteric Fracture. Not all trochanteric fractures are accompanied by greater trochanteric (GT) fractures. (**A**) The X-ray image of a patient with a trochanteric fracture and (**B**) the short-axis (SAX) view ultrasound image of the GT region from the same patient are shown. A GT fracture is visible on the X-ray and is also confirmed on sonography. If a tear is observed in this region, it is unlikely to have occurred iatrogenically. In another patient with a trochanteric fracture, (**C**) the X-ray image and (**D**) the SAX view ultrasound image show no evidence of a GT fracture on sonography. If a gluteal tendon tear is observed here, it suggests that the tear is of iatrogenic origin.

Findings through ultrasound were classified according to following criteria: (1) If hypoechoic lesions or fibrillar disruptions involved less than 25% of the tendon thickness, or if tendon thickening or osteophytes were observed, the condition was classified as tendinosis [39]. (2) If hypoechoic lesions or fibrillar disruptions involved 25% or more of the tendon thickness, the condition was classified as a tendon tear [39]. (3) Regardless of the presence of acoustic shadowing, if hyperechoic foci were seen within the tendon, the condition was classified as calcification [40]. (4) An interruption of the cortical contour of the bone was classified as a fracture [41] (Figure 4). (5) A tendon tear identified on a facet of the greater trochanter without an accompanying fracture at the same location, presumed to have occurred during the intramedullary nailing procedure was classified as a iatrogenic injury. (6) A tendon tear observed in conjunction with a fracture at the same facet, considered to have resulted from the initial traumatic event was classified as a trauma-associated injury (Figure 5). All findings were cross confirmed by the senior clinician.

Although the location of the incision site is distant from the apex of the GT (Figure 4), which reduces the risk of wound contamination, aseptic chlorhexidine gel was used as the coupling gel during the ultrasound examination to minimize complications, such as infections [42,43]. The dressing was reapplied after the ultrasound.

### 2.6. Statistical Analysis

Descriptive statistics were used to summarize patient demographics, fracture characteristics, and ultrasound findings. Continuous variables such as age were expressed as means and standard deviations, while categorical variables including sex, fracture type, and lesion frequency were presented as counts and percentages.

To evaluate the association between implant type and the incidence of iatrogenic Gme tendon tear, a subgroup analysis was performed using Fisher’s exact test. A *p*-value less than 0.05 was considered statistically significant.

## 3. Results

The frequency of each type of lesion was assessed on both the unaffected and affected sides. If the same type of lesion was observed on different facets, it was counted as a single case (e.g., if tendinosis was found in both the A and L facets in a single patient, it was counted as one case of tendinosis, not two). If no lesions were observed in any of the three facets, the case was classified as normal.

On the affected side, 7 cases of calcification (11%), 48 cases of fracture (79%), 35 cases of tears (57%), and 10 cases of tendinosis (16%) were observed. On the unaffected side, lesions were identified in 92% of patients, with 5 normal cases (8%), 39 cases of tendinosis (64%), and 30 cases of calcification (49%), making tendinosis the most commonly observed lesion (Table 2).

**Figure 6:**
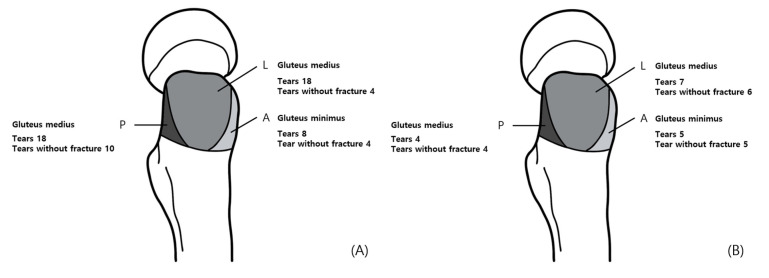
Three facets of greater trochanter and incidence of pathology. Incidence of pathology: (**A**) in patients with trochanteric fractures, (**B**) in patients with without trochanteric fractures.

In patients with trochanteric fractures, tendon tears on the affected side were observed in 25 patients: 8 cases in the A facet, 18 cases in the L facet, and 18 cases in the P facet. Among these, tendon tears without concurrent fractures in the same facet occurred in 4 cases in the A facet, 4 cases in the L facet, and 10 cases in the P facet. After excluding duplicates, a total of 11 cases of tendon tears without fractures were identified (Figure 6A).

In 13 patients without trochanteric fractures, tendon tears were observed on the affected side in 11 patients: five cases in the A facet, seven cases in the L facet, and four cases in the P facet. Among these, in cases without concurrent fractures in the same facet, there were five cases in the A facet, six cases in the L facet, and four cases in the P facet. After excluding duplicates, a total of 10 cases of tendon tears without fractures on the affected side were identified (Figure 6B).

In patients with trochanteric fractures, 11 cases of Gme tendon tears without accompanying fractures were identified, while 14 cases were associated with fractures. In the affected side of patients without trochanteric fractures, nine cases of Gme tendon tears without fractures were observed, whereas only one case involved both a tendon tear and a fracture.

On the affected side, lesions in the Gmi included 33 normal cases (54%), 10 cases of tendinosis (16%), 2 cases of calcification (3%), 13 cases of tears (21%), and 7 cases of fracture (11%). In the Gme on the affected side, there were no normal cases (0%), 4 cases of tendinosis (7%), 6 cases of calcification (10%), 31 cases of tears (51%), and 47 cases of fracture (77%). On the unaffected side, lesions in the Gmi were as follows: 24 normal cases (39%), 23 cases of tendinosis (38%), and 17 cases of calcification (28%). In the Gme on the unaffected side, there were 6 normal cases (10%), 37 cases of tendinosis (61%), and 28 cases of calcification (46%) (Table 3).

In a subgroup analysis, implant type was associated with varying rates of iatrogenic Gme injury. Long Gamma nails showed the highest injury rate (5 out of 7 cases, 71%), followed by GS Hip nails (12 out of 43, 28%) and Gamma 3 nails (2 out of 9, 22%). The difference between Long Gamma and GS Hip nails was statistically significant (*p* = 0.037).

## 4. Discussion

This study is the first to evaluate hip abductor injury after IM nailing surgery. Although similar studies have been conducted previously, they did not assess the injury immediately postoperatively, but instead evaluated the gluteus tendon in the surviving population [7]. The evaluation in the surviving population could lead to survivor bias, as only patients with well-preserved gluteal tendons are likely to survive [44]. In this study, injuries were assessed before discharge, enabling the determination of whether the gluteus tendon damage occurred during the surgery or was associated with a concurrent trochanteric fracture. Additionally, since tendon injuries in proximal femur fractures can be difficult to distinguish from degenerative changes over time, this study assessed both degenerative changes on the contralateral side and acute-phase injuries on the same day.

Previous studies have examined muscle or tendon damage associated with intramedullary nail entry points, either through cadaveric models or postoperative imaging in clinical settings [7,11]. While these studies provided valuable insights into the evaluation of the entry point, they did not classify or assess the Gmi and Gme in relation to the A, L, and P regions of the GT facet. Additionally, methods to account for volume averaging effects were not proposed, and both short-axis and long-axis evaluations of the tendon insertion sites were not performed, potentially leading to an underestimation of enthesis damage. In this study, we conducted examinations on all patients before discharge and used both long-axis and short-axis views to minimize underestimation, striving for a more accurate diagnosis.

Although intramedullary nailing remains the gold standard for proximal femur fracture fixation, our study identified iatrogenic Gme tendon tears in 20 patients (33%) and Gmi tears in a smaller subset. Given the anatomical proximity of the Gme to the trochanteric fossa, this muscle was initially expected to sustain the highest rate of iatrogenic injury. However, the actual incidence was lower than anticipated, and Gmi involvement was also observed. These findings are consistent with previous reports suggesting that soft tissue damage during fracture reduction may contribute to tendon injuries [45,46]. Such damage, particularly in the setting of closed reduction and reaming, may compromise early postoperative mobility and rehabilitation [47]. Additionally, when Gme tears are associated with fractures, the healing process may be hindered by the presence of the intramedullary device at the tendon’s attachment site, potentially impacting long-term functional outcomes.

Furthermore, our study was primarily designed to evaluate the prevalence of abductor tendon injuries using postoperative ultrasound; nevertheless, we performed an exploratory subgroup analysis comparing injury rates across different implant types. Interestingly, Long Gamma nails showed the highest rate of iatrogenic Gme injuries, despite having a slightly smaller proximal diameter than GS Hip nails. This counterintuitive finding suggests that nail design characteristics, such as entry angle, proximal geometry, or reaming trajectory, may play a more substantial role than diameter alone. While these findings are preliminary, they raise important questions that warrant further biomechanical and clinical investigation in future studies.

Ultrasound evaluation of the contralateral side revealed degenerative changes, such as calcification and tendinosis, in most cases. These changes could limit the patient’s mobility and contribute to deficits in proprioception [48,49,50]. Therefore, the causes of proximal femur fractures should not be limited to osteoporosis or sarcopenia alone [51,52]. Identifying such degenerative lesions and implementing isometric or proprioceptive neuromuscular facilitation (PNF) exercises within the community could potentially aid in fall prevention [53,54]. Applying these strategies to patients who have undergone hip fracture surgery may reduce recurrence of fracture and assist in return to daily activities [55]. Additionally, performing ultrasound evaluations on healthy individuals of same age group and comparing the findings could help identify age-related changes. Promoting exercise in individuals above a certain age threshold could reduce socioeconomic costs [56].

Ultrasound evaluation of the hip is typically performed using a curvilinear probe [57]. However, in this study, we used a linear probe. While the curvilinear probe provides better penetration for visualizing deep structures, it has limitations in accurately assessing muscle and tendon damage. In contrast, the linear probe allows for more detailed imaging for superficial tissues, although offering lower penetration for deeper tissues [58]. In this study, we first performed a preliminary assessment to confirm the probe’s penetration capabilities. The patients were positioned in the lateral position to minimize tissue depth, and a 12 L probe was used, which allowed simultaneous observation of both superficial and deep tissues. Additionally, the appropriate preset for deep tissue imaging was applied to offset the limitations of the linear probe. This approach enabled accurate evaluation of muscle and tendon injuries, which were the primary focus of this study.

This study has several limitations, primarily related to methodological bias. First, as there is currently no standardized sonographic definition of tendon lesions around the hip joint, we adopted ultrasound criteria used for supraspinatus tendon evaluation. While this approach allowed for practical assessment, it limited our ability to differentiate between partial- and full-thickness tears, or to grade tear size (e.g., small, medium, large). As no complete tears were observed in our cohort, all lesions were conservatively classified as partial tears [59,60].

Second, the lack of preoperative ultrasound examination hindered our ability to distinguish iatrogenic injury from trauma-related damage. This was due to patient cooperation issues caused by pain at the fracture site, and concerns about prolonging anesthesia time if preoperative evaluation was conducted intraoperatively.

Third, to address the relatively small sample size (n = 61), we included assessments of the contralateral, non-operated side. However, the absence of an age-matched external control group may limit the generalizability of our findings. And the fact that all surgeries were performed by a single surgeon at a single institution may further restrict the external validity of the study, despite the benefit of procedural consistency this setting provides.

## 5. Conclusions

Minimally invasive osteosynthesis, such as intramedullary (IM) nailing, is recognized as the gold standard treatment for femur fractures. However, in our study, sonographic evaluations indicated an incidence of hip abductor injury exceeding 57% following IM nailing. Therefore, postoperative rehabilitation for femur fracture patients treated with IM nailing should focus on the healing and strengthening of the Gme to promote earlier ambulation. Degeneration of the abductor muscles, such as tendinosis or calcification, was observed in approximately 92% of unaffected limbs. This suggests that degeneration of the abductor muscles may be a risk factor for falls and subsequent hip fractures.

## Figures and Tables

**Table 1:** Patient Characteristics at Baseline.

Characteristics	Patients, No. (%) (N = 61)
Age, mean (SD), y	79 (12)
Gender	
Female	46 (75%)
Male	15 (25%)
Diagnosis ^†,a^	
Trochanteric fracture	48 (79%)
Mid-shaft fracture	5 (8%)
Subtrochanteric fracture	4 (7%)
Femur neck stress fracture	1 (2%)
Proximal shaft fracture	1 (2%)
Mid-shaft peri-implant fracture	1 (2%)
IM nail removal	1 (2%)

Abbreviation: IM, intramedullary. ^†^ Fracture classifications: Garden (femoral neck), AO/OTA (intertrochanteric, subtrochanteric, shaft); ^a^ Rounding to the nearest whole number at the first decimal place may result in the total percentage not equaling 100%.

**Table 2:** Frequency of occurrence according to type of lesion.

Type of Lesion	Unaffected Side (N = 61) ^a^	Affected Side (N = 61) ^b^
Normal	5 (8%)	0 (0%)
Tendon tear	0 (0%)	35 (57%)
Tendinosis	39 (64%)	10 (16%)
Calcification	30 (49%)	7 (11%)
Fracture	0 (0%)	48 (79%)

^a,b^ Since lesions may overlap within each muscle, the total percentage does not add up to 100%.

**Table 3:** Frequency of occurrence according to types of lesions in the gluteal muscles.

Type of Lesion	Location of Lesion	
	Gmi (N = 61) ^a^	Gme (N = 61) ^b^
Unaffected side		
Normal	24 (39%)	6 (10%)
Tendinosis	23 (38%)	37 (61%)
Calcification	17 (28%)	28 (46%)
Tendon tear	0 (0%)	0 (0%)
Fracture	0 (0%)	0 (0%)
Affected side		
Normal	33 (54%)	0 (0%)
Tendinosis	10 (16%)	4 (7%)
Calcification	2 (3%)	6 (10%)
Tendon tear	13 (21%)	31 (51%)
Fracture	7 (11%)	47 (77%)

Abbreviations: Gmi, gluteus minimus; Gme, gluteus medius. ^a,b^ Since lesions may overlap within each muscle, the total percentage does not add up to 100%.

## Data Availability

The raw data supporting the conclusions of this article will be made available by the authors on request.
